# CXCR1/2 Antagonism Is Protective during Influenza and Post-Influenza Pneumococcal Infection

**DOI:** 10.3389/fimmu.2017.01799

**Published:** 2017-12-13

**Authors:** Luciana P. Tavares, Cristiana C. Garcia, Marina G. Machado, Celso M. Queiroz-Junior, Adeline Barthelemy, François Trottein, Marilda M. Siqueira, Laura Brandolini, Marcello Allegretti, Alexandre M. Machado, Lirlândia P. de Sousa, Mauro M. Teixeira

**Affiliations:** ^1^Laboratóriode Imunofarmacologia, Departamento de Bioquímica e Imunologia, Instituto de Ciencias Biologicas (ICB), Universidade Federal de Minas Gerais, Belo Horizonte, Brazil; ^2^Laboratório de Vírus Respiratórios e do Sarampo, Instituto Oswaldo Cruz (Fiocruz), Rio de Janeiro, Brazil; ^3^Departamento de Análises Clínicas e Toxicológicas, Faculdade de Farmácia, Universidade Federal de Minas Gerais, Belo Horizonte, Brazil; ^4^Departamento de Morfologia, Instituto de Ciencias Biologicas (ICB), Universidade Federal de Minas Gerais, Belo Horizonte, Brazil; ^5^Centre d’Infection et d’Immunité de Lille, INSERM U1019, CNRS UMR 8204, University of Lille, CHU Lille, Institut Pasteur de Lille, Lille, France; ^6^R&D Department, Dompé Farmaceutici s.p.a., L’Aquila, Italy; ^7^Centro de Pesquisas René Rachou, Fundação Oswaldo Cruz, Belo Horizonte, Brazil

**Keywords:** inflammation, immunomodulation, CXCR1/2, influenza A, pneumococcus, secondary infection

## Abstract

**Rationale:**

Influenza A infections are a leading cause of morbidity and mortality worldwide especially when associated with secondary pneumococcal infections. Inflammation is important to control pathogen proliferation but may also cause tissue injury and death. CXCR1/2 are chemokine receptors relevant for the recruitment of neutrophils. We investigated the role of CXCR1/2 during influenza, pneumococcal, and post-influenza pneumococcal infections.

**Methods:**

Mice were infected with influenza A virus (IAV) or *Streptococcus pneumoniae* and then treated daily with the CXCR1/2 antagonist DF2162. To study secondary pneumococcal infection, mice were infected with a sublethal inoculum of IAV then infected with *S. pneumoniae* 14 days later. DF2162 was given in a therapeutic schedule from days 3 to 6 after influenza infection. Lethality, weight loss, inflammation, virus/bacteria counts, and lung injury were assessed.

**Results:**

CXCL1 and CXCL2 were produced at high levels during IAV infection. DF2162 treatment decreased morbidity and this was associated with decreased infiltration of neutrophils in the lungs and reduced pulmonary damage and viral titers. During *S. pneumoniae* infection, DF2162 treatment decreased neutrophil recruitment, pulmonary damage, and lethality rates, without affecting bacteria burden. Therapeutic treatment with DF2162 during sublethal IAV infection reduced the morbidity associated with virus infection and also decreased the magnitude of inflammation, lung damage, and number of bacteria in the blood of mice subsequently infected with *S. pneumoniae*.

**Conclusion:**

Modulation of the inflammatory response by blocking CXCR1/2 improves disease outcome during respiratory influenza and pneumococcal infections, without compromising the ability of the murine host to deal with infection. Altogether, inhibition of CXCR1/2 may be a valid therapeutic strategy for treating lung infections caused by these pathogens, especially controlling secondary bacterial infection after influenza.

## Introduction

The lungs are composed of a myriad of tree-like ramifications that end in intensively vascularized alveoli. The mucosal surface of the lung is incredibly large (90 m^2^) and is exposed daily to a high number of particles and microorganisms including pathogens (1). Therefore, a great number of physical and biological barriers, including the innate immune system, protect the lungs from a possible infection. Pro-inflammatory cytokines and chemokines are produced by resident immune cells and lung epithelial cells promoting recruitment of neutrophils and the onset of inflammation. This is important to control dissemination and proliferation of microorganisms. However, the uncontrolled inflammatory response triggered by infection may also lead to increased lung damage, morbidity, and mortality (2).

Influenza A virus (IAV) is a respiratory pathogen of major worldwide relevance, causing 3 to 5 million of severe illness and more than 300,000 deaths during epidemics.^1^ Secondary bacterial infections contribute greatly to the increased mortality and morbidity during seasonal and also pandemics influenza. It is estimated that bacterial coinfections are responsible for approximately 25% of influenza-related deaths (3). Among different bacteria that cause secondary infection after the flu, *Streptococcus pneumoniae* stands out as one of the most important pathogens (4). Indeed, *S. pneumoniae* is considered as a primary cause of mortality during seasonal flu (5) and a leading cause of community-acquired pneumonia among children and adults, mostly among those who had the flu previously (6). Despite the availability of antibiotics, the incidence and lethality of secondary pneumococcal infections after flu is still high. In fact, during IAV and pneumococcus coinfection, treatment with antibiotics may cause bacteria lysis, excessive stimulation of the immune system, and massive recruitment of neutrophils, events that may lead to intense tissue damage and mortality (7).

Neutrophils are one of the first cells recruited into the lungs during IAV and pneumococcal infections (8–10). Once the microorganisms reach the lung epithelium, they are recognized by immune and non-immune cells leading to secretion of chemokines such as CXCL8 (CXCL1/CXCL2 in mice) (8). These chemokines act through its receptors CXCR1 and CXCR2 expressed by different cell types such as monocytes, CD8^+^ T cells, natural killer cells, and neutrophils. In neutrophils, activation of CXCR1 and CXCR2 leads to chemotaxis, release of granule enzymes, and production of reactive oxygen species (11). These events are very important to control virus or bacteria proliferation and dissemination, but overwhelming activation of neutrophils can be detrimental for the host as it can lead to intense lung injury. This is true for both IAV and pneumococcus infections, as an intense influx of highly activated neutrophils are associated with disease severity (12, 13). Therefore, different strategies to control the inflammatory response during respiratory infections have been suggested as an attempt to reduce disease magnitude (2, 13).

Here, we investigated the effects of CXCR1/2 antagonism using the compound DF2162 (14) during IAV and pneumococcus primary infections. The results show that prophylactic treatment with DF2162 did not have a detrimental role during primary infections, instead it decreased neutrophil recruitment, morbidity, and mortality associated with both IAV and *S. pneumoniae* infections. Therefore, we decided to use it therapeutically as a strategy to control disease caused by a pneumococcal infection that followed infection by IAV. Treatment with the compound from days 3 to 6 after IAV infection prevented lung damage and morbidity caused by a subsequent pneumococcal infection. Despite the reduction of inflammation, treatment with DF2162 did not reduce the host ability to control either single or secondary infection. Therefore, modulation of inflammation during IAV infection by CXCR1/2 antagonists, such as DF2162, may be an interesting strategy to treat the flu and decrease the morbidity associated with secondary bacterial infections.

## Materials and Methods

### Mice

Male C57BL/6J mice (8–12 weeks old) were obtained from the Central Animal Facility from Universidade Federal de Minas Gerais (CEBIO UFMG/Brazil) and were maintained with free access to commercial chow and water. All procedures described had prior approval of the local animal ethics committee (CETEA/UFMG 13/2010 and 381/2015).

### Bacterial and Virus Strains

*Streptococcus pneumoniae* (ATCC 6303 serotype 3) infection stocks were prepared as described previously (13). The inocula were confirmed by plating of bacterial suspension.

Influenza A/WSN/33 H1N1—herein called IAV or Flu—was grown in MDCK (Madin–Darby Canine Kidney) cultured cells as described (15). Before infection, the stocks were thawed on ice and diluted in sterile PBS.

### Infection of Mice

For IAV and *S*. *pneumoniae* single infections, mice were anesthetized with 60 mg/kg of ketamine and 4 mg/kg of xylazine and instilled intranasally with 1 × 10^4^ PFU (about LD_50_ inoculum) or 1 × 10^6^ PFU (lethal inoculum) of IAV or 1 × 10^4^ CFU of *S. pneumoniae* diluted in PBS. For the secondary pneumococcal infection model, anesthetized mice were infected with 500 PFU of IAV and after 14 days of viral infection, mice were anesthetized with isofluorane and then infected with 10^3^ CFU of *S*. *pneumoniae*. Control mice received PBS intranasally (Mock infection).

### Treatment Protocol

To evaluate the effect of CXCR1/2 antagonism during respiratory infections, mice were treated with the non-competitive allosteric CXCR1/2 antagonist, DF2162 (100 µl—10 mg/kg) diluted in 0.1% carboxymethylcellulose (CMC) by oral gavage (Labsynth^®^, Sao Paulo, Brazil). Vehicle-treated animals received 100 µl of 0.1% of CMC only (16). This dose and schedule of administration have been shown to cause significant inhibition of neutrophil influx in other models and are consistent with the long half-life of the molecule (14, 16).

For the single IAV infection, infected mice (10^4^ PFU) were treated twice a day for 5 days from the day of infection. Mice were euthanized after 5 days of infection to access inflammation, virus titer, and lung damage. Weight loss was also accompanied.

For the pneumococcal single infection, infected mice (1 × 10^5^ CFU) were treated after 6 h of infection and then after 12, 24, and 36 h. After 48 h of infection, mice were euthanized for evaluation of lung injury, bacteria counts, and inflammation. For the lethality experiments, mice were treated twice a day for 2 days and accompanied for 10 days.

Finally, for the secondary pneumococcal infection experiments, mice were infected with 500 PFU of IAV and treated from day 3 to 6 of infection (twice a day). After 14 days of IAV infection, mice were infected with 10^3^ CFU *S. pneumoniae*. Lethality and weight loss were accompanied. Mice were euthanized after 16 days of IAV infection (2 days after pneumococcal infection) for analysis of lung damage, inflammation, and bacteria counts in the airways and blood.

### Bronchoalveolar Lavage (BAL) and Tissue Extraction

At indicated time points, mice were euthanized with a lethal dose of ketamine/xylazine (180 and 15 mg/kg, respectively), blood was collected for bacteria counts and antibodies levels (serum) and BAL was performed. For that, mice trachea was exposed, a 1.7-mm catheter was inserted and two aliquots of 1 ml of PBS were flushed three times into the brochoalveolar compartment to recover the leukocytes and bacteria in the airways of mice (15). 100 µl of BAL fluid were plated in blood agar for bacterial counts. After centrifugation, the pellet of cells was used to total and differential cell counts. Cytocentrifuge preparations (Shandon III) stained with May–Grunwald–Giemsa were used for differential counts of leukocytes, based on morphological criteria. Each slide was counted three times and the percentage was used to calculate the absolute number of each leukocyte type. The counts were performed by a researcher blinded to the treatments. BAL fluid supernatants were used for cytokines (IL-12p40, IL-10, TNF-α, IL-6, CXCL1, and CXCL2) measurements by ELISA according to the manufacturer’s instructions (R&D Systems, USA) and total protein quantification using the Bradford assay (Biorad). The right lung of mice was collected for indirect quantification of neutrophil recruitment into the tissue (myeloperoxidase assay—MPO) and for virus titration. The left lobe of the lungs was fixed in formalin for further histological examination.

### Flow Cytometry

Mice were infected with 500 PFU of IAV and treatment with DF2162 or vehicle started 3 days after infection (twice a day until day 6). At day 14 post-infection, mice were euthanized and the lungs were collected to access T cell number and subtypes. Lungs were homogenized, and leukocytes were labeled with appropriate dilutions of Pacific Blue-conjugated anti-CD45, PerCP-conjugated anti-CD3, APC-conjugated CD8, and APC-Cy7-conjugated CD4. Events were acquired using a LSRFortessa cytometer (Becton Dickinson Biosciences, Rungis, France) running FACSDiva software and were then analyzed with the FlowJo software.

### *Ex Vivo* Stimulation of Macrophages

Aiming to access the production of CXCL1 by macrophages after IAV infection, mice were infected with 500 PFU of IAV and treated with DF2162 or the vehicle of the drug (0.1% of CMC) twice a day at days 3–6 of IAV infection. At day 14, mice were euthanized, BALF was performed, cells were counted, and 10^5^ cells were plated in 96-well culture plates. After 3 h of incubation at 37°C and 5% of CO_2_, the cells were washed with DMEM and stimulated with live *S. pneumoniae* (MOI 1:10) for 1 h. Later, the cells were washed and incubated overnight at 37°C and 5% of CO_2_. Cell culture supernatants were collected for the analysis of CXCL1 production by ELISA, per the manufacturer’s instructions.

### Lung MPO

Fifty milligrams of lung tissue were homogenized in a buffered solution containing antiproteases, as previously described (16). MPO levels were accessed using 25 μl of the supernatant of the homogenized sample and 25 µl of a solution of 1.6 mM of 3,39-5,59-tetramethylbenzidine (TMB; Sigma—dissolved in dimethyl sulfoxide) and 0.01 mM of H_2_O_2_, diluted in phosphate buffer (pH 5.4) containing HTAB (16).

### Virus Quantification (Plaque Assay)

For virus titrations, lungs collected in sterile conditions were weighted and homogenized in sterile cold PBS. Serial dilutions of samples were incubated in MDCK cells monolayers for 1 h, covered with agarose for 72 h as previously described (15). The number of plaque forming units was expressed per gram of lung.

### Hemagglutination Inhibition (HI)

Influenza A/WSN/33 H1N1 virus was tittered for hemagglutination activity (HA) by incubating twofold virus dilutions with 0.75% of guinea pig red blood cells (RBCs). Sera from mock, IAV, and *S. pneumoniae*-infected mice collected 16 days after IAV infection were treated with receptor destroying enzyme (RDE) for 16 h and then treated with RBC to remove unspecific HA. RDE/RBC treated sera were diluted from 10 to 1,280 and incubated with 8 unities of HA of WSN per 50 µl in U shaped 96-well plates. Then, 0.75% RBC was added to all wells. HI titer was assumed as the last serum dilution where 100% of HI was detected.

### Histological Analyses

To access lung damage followed by IAV and pneumococcus infections, fixed left lobes of the lungs were gradually dehydrated in ethanol and embedded in paraffin. 4-µm sections were cut and stained with H&E for examination under light microscopy. The histopathological score was performed by a pathologist blinded to the experimental groups and evaluated airway, vascular and parenchymal inflammation, epithelial damage, and general neutrophilic inflammation, in an 18-points score (15).

### Statistical Analyses

Statistics were performed using GraphPad Prism 4.0. One-way ANOVA, followed by Newman–Keuls post-test was used to compare more than two groups and unpaired *t*-test was used for comparisons between two groups. The survival curves were analyzed by Long-rank test and the weight loss curves were compared using analyses of area under the curve. Results with *P* < 0.05 were considered statistically significant.

## Results

### IAV Infection Increases the Levels of CXCL1 and CXCL2 and Leads to an Increased Influx of Neutrophils into the Airways and Lungs of Mice

**Figure 1:**
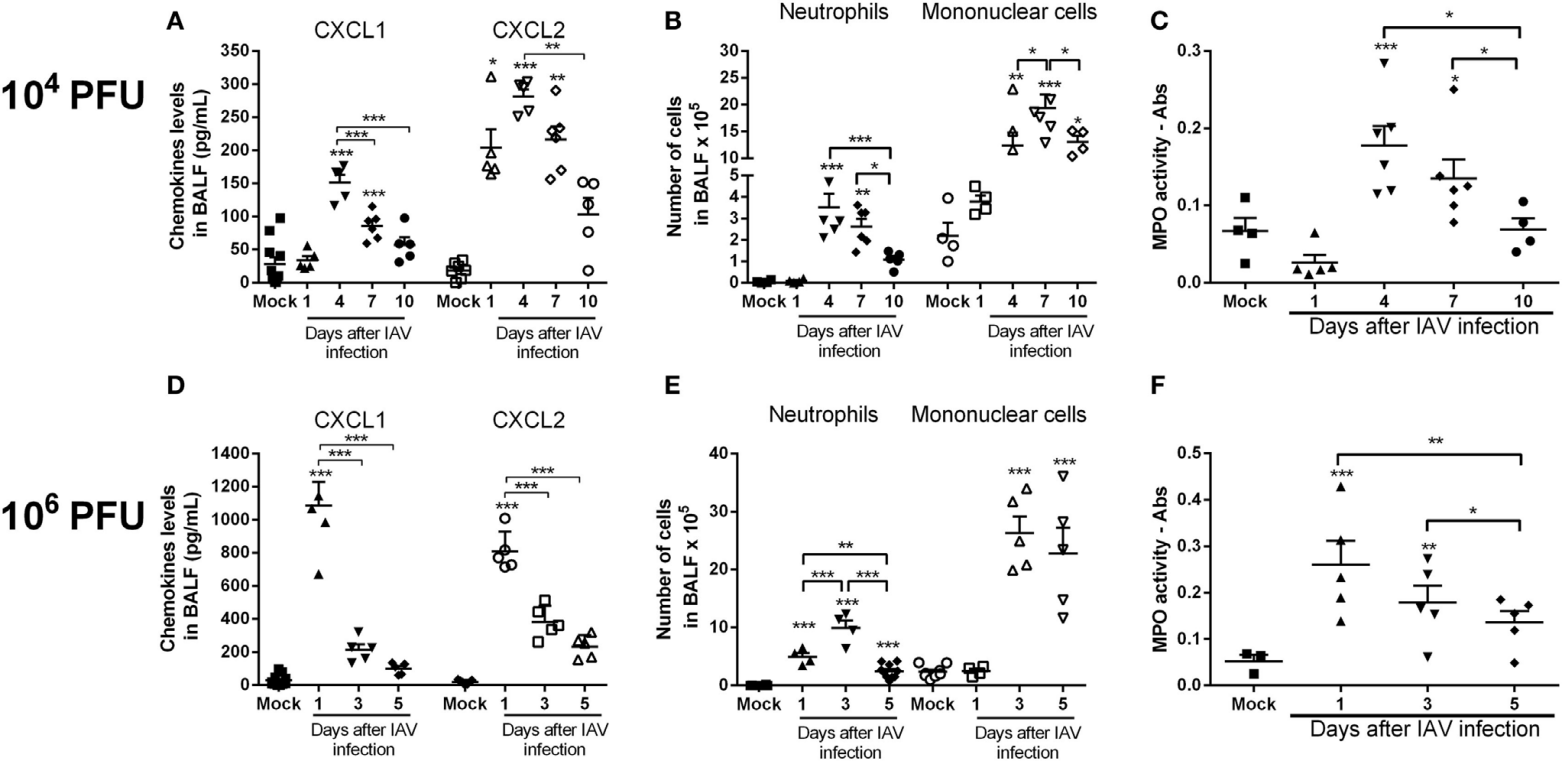
Kinetics of inflammatory responses triggered by influenza A virus (IAV) infection. Mice were infected with IAV (10^4^ and 10^6^ PFU) or instilled with PBS (Mock) and after 1, 3, 4, 5, 7, and 10 days of infection were euthanized. Levels of the chemokines CXCL1 and CXCL2 **(A,D)**, number of neutrophils and mononuclear cells in the airways **(B,E)** and myeloperoxidase assay (MPO) levels in lungs **(C,F)** were evaluated at different times after infection (*n* = 5–6 mice per group—representative of two experiments). Results are expressed as the number of cells, levels of cytokines (pg/ml), absorbance or percentage of initial weight, and are shown as the mean ± SEM. *P < 0.05; **P < 0.01; ***P < 0.001, when compared with Mock mice or indicated groups.

To investigate neutrophil infiltration and levels of the chemokines CXCL1 and CXCL2 after lethal or sublethal IAV infection, mice were infected with 1 × 10^6^ PFU (lethal inoculum) or 1 × 10^4^ PFU (about LD_50_ inoculum) of the virus, respectively. After 1, 4, 7, and 10 days for the lower inoculum or after 1, 3, and 5 days for the higher inoculum, BAL and lungs were collected. Levels of both chemokines in the airways peaked 4 days after sublethal IAV infection and decreased thereafter (Figure 1A). Infection with the lethal inoculum resulted in faster and higher production of chemokines in the airways (Figure 1D). The increased production of CXCL1 and CXCL2 was associated with a massive influx of neutrophils into the airways and lungs (as assessed by MPO activity) and it was inoculum dependent (Figures 1B,C,E,F). The number of mononuclear cells was also increased after IAV infection (Figures 1B,E).

### CXCR1/2 Antagonism Lowers Influenza-Induced Pulmonary Inflammation

**Figure 2:**
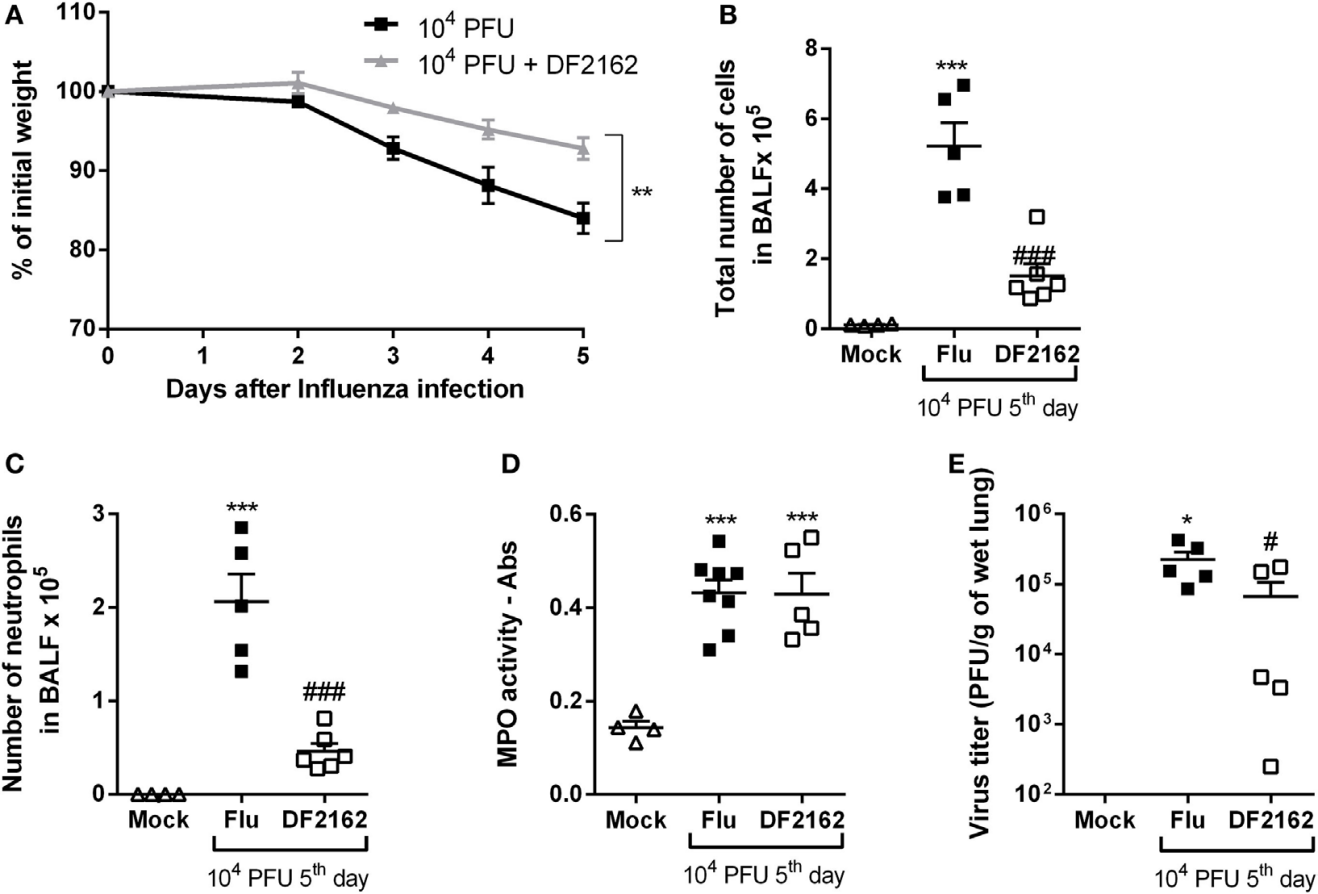
CXCR1/2 antagonism decreases the inflammatory responses during influenza A virus (IAV) infection and protects mice from morbidity. Mice were infected with 10^4^ PFU of IAV and treated with DF2162 (10 mg/kg) twice a day during the first 5 days of infection or with the drug vehicle (carboxymethylcellulose 0.1% in PBS). Control animals were instilled intranasally with PBS (Mock). Weight loss **(A)**, number of total leukocytes **(B)**, and neutrophils **(C)** in the airways or myeloperoxidase assay (MPO) levels in lungs **(D)** and the virus counts in the lungs **(E)** were evaluated after 5 days of infection (*n* = 5–6 mice per group—representative of two independent experiments). Data are presented as the mean ± SEM. *P < 0.05; **P < 0.01, and ***P < 0.001, when compared with Mock group and ^#^*P* < 0.05 and ^###^*P* < 0.001 when compared with vehicle group (Flu).

**Figure 3:**
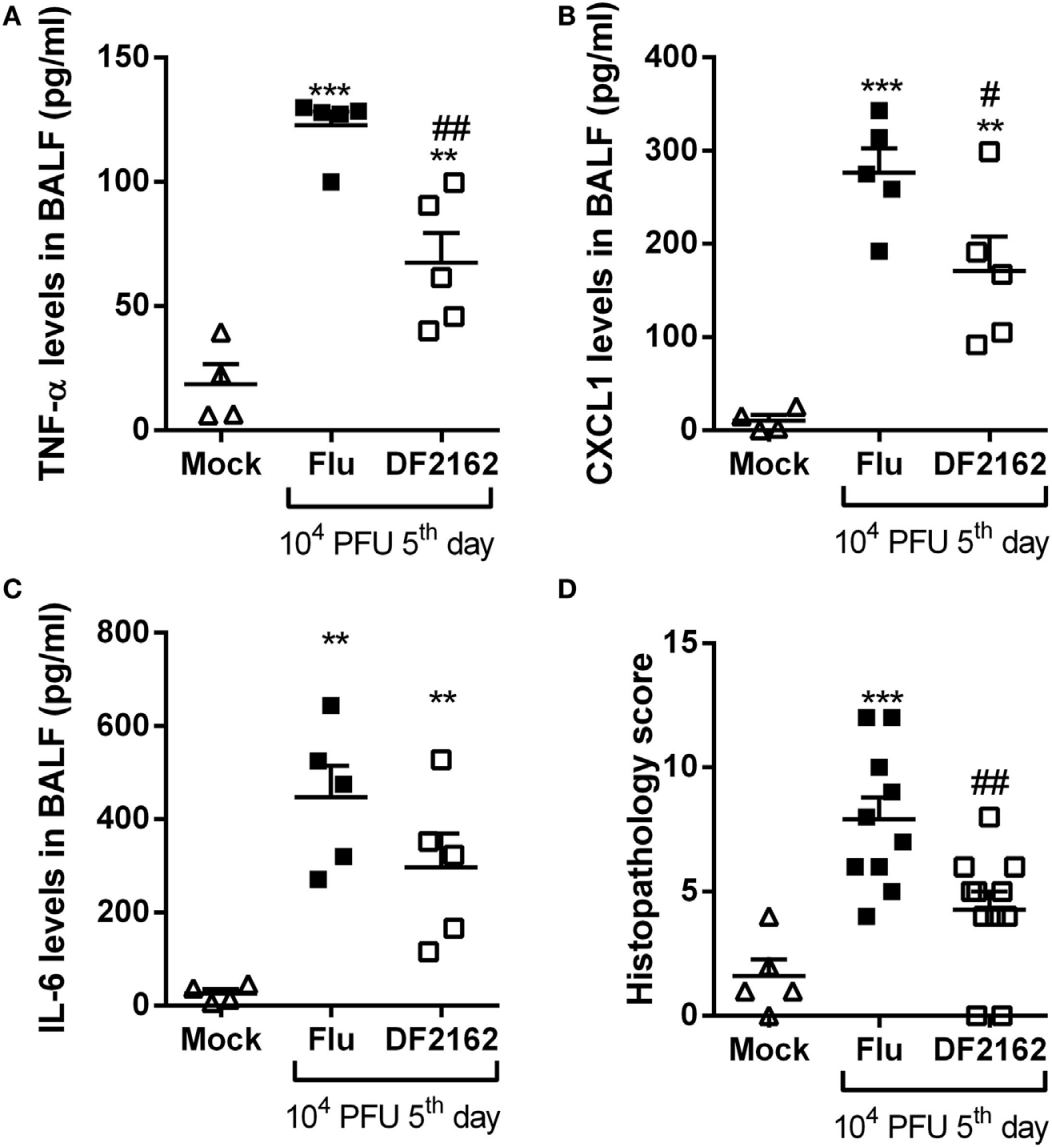
Levels of pro-inflammatory cytokines and lung damage are reduced after DF2162 treatment. Mice were infected with 10^4^ PFU of influenza A virus and treated with DF2162 (10 mg/kg) twice a day during the first 5 days of infection or with the drug vehicle (carboxymethylcellulose 0.1% in PBS). Control animals were instilled intranasally with PBS (Mock). Levels of TNF-α **(A)**, CXCL1 **(B)**, and IL-6 **(C)** in the airways of mice were measured. Histological analyses were performed and the histopathological score is presented in **(D)**—maximal of 18 points (airway, vascular, parenchymal inflammation, neutrophilic infiltration, and epithelial injury). The results are presented as mean ± SEM (*n* = 5–6 mice per group representative of two independent experiments). **P < 0.01 and ***P < 0.001 when compared to Mock group; ^#^*P* < 0.05 and ^##^*P* < 0.01 when compared to Vehicle group.

As the overwhelming neutrophilic inflammation and intense CXCL1 and CXCL2 production seemed to be associated with the severity of IAV infection, we wondered if antagonism of the receptors for CXCL1 and CXCL2 would impact the course of infection. Of note, there is much evidence that mice express the human homolog of CXCR2, the chemokine receptor for CXCL-8, and other related chemokines (17). The murine receptor homolog for human CXCR1 has also been reported but its role in murine models of inflammation is poorly known (18). Despite this potential controversy, the drug used, DF2162, has been reported to block both chemokine receptors in humans (17) and CXCL1-mediated migration of murine neutrophils (unpublished data). To investigate the role of CXCR1/2 during influenza infection, mice were infected with 1 × 10^4^ PFU of IAV and then treated twice a day (from day 0—at the time of the infection—to day 5 post-infection) with DF2162 at a dose that efficiently decreased neutrophil numbers in the lungs of mice in another model of inflammation (16). Treatment with DF2162 decreased morbidity, as seen by the reduction of weight loss (Figure 2A). Drug treatment also decreased several parameters of the inflammatory response, including number of total leukocytes recruited into the airways (Figure 2B), including neutrophils (Figure 2C), and levels of the pro-inflammatory cytokines TNF-α and CXCL1 (Figures 3A,B). Treatment with DF2162 did not reduce levels of MPO in the lungs of infected mice (Figure 2D) or the levels of IL-6 (Figure 3C). Interestingly, viral loads in the lungs of DF2162-treated mice were only slightly reduced, as compared with vehicle-treated animals (Figure 2E). In addition, treatment with DF2162 reduced the lung injury associated with IAV infection (Figure 3D). Histological analysis showed more preserved areas of lung, with reduced bronchiolar and vascular inflammation in the lungs of treated animals (Figure S1 in Supplementary Material). Therefore, CXCR1/2 antagonist does not impact the course of IAV infection itself, but reduces associated inflammation and consequently the morbidity.

### CXCR1/2 Antagonism Reduces Pneumonia during *S. pneumoniae* Infection

**Figure 4:**
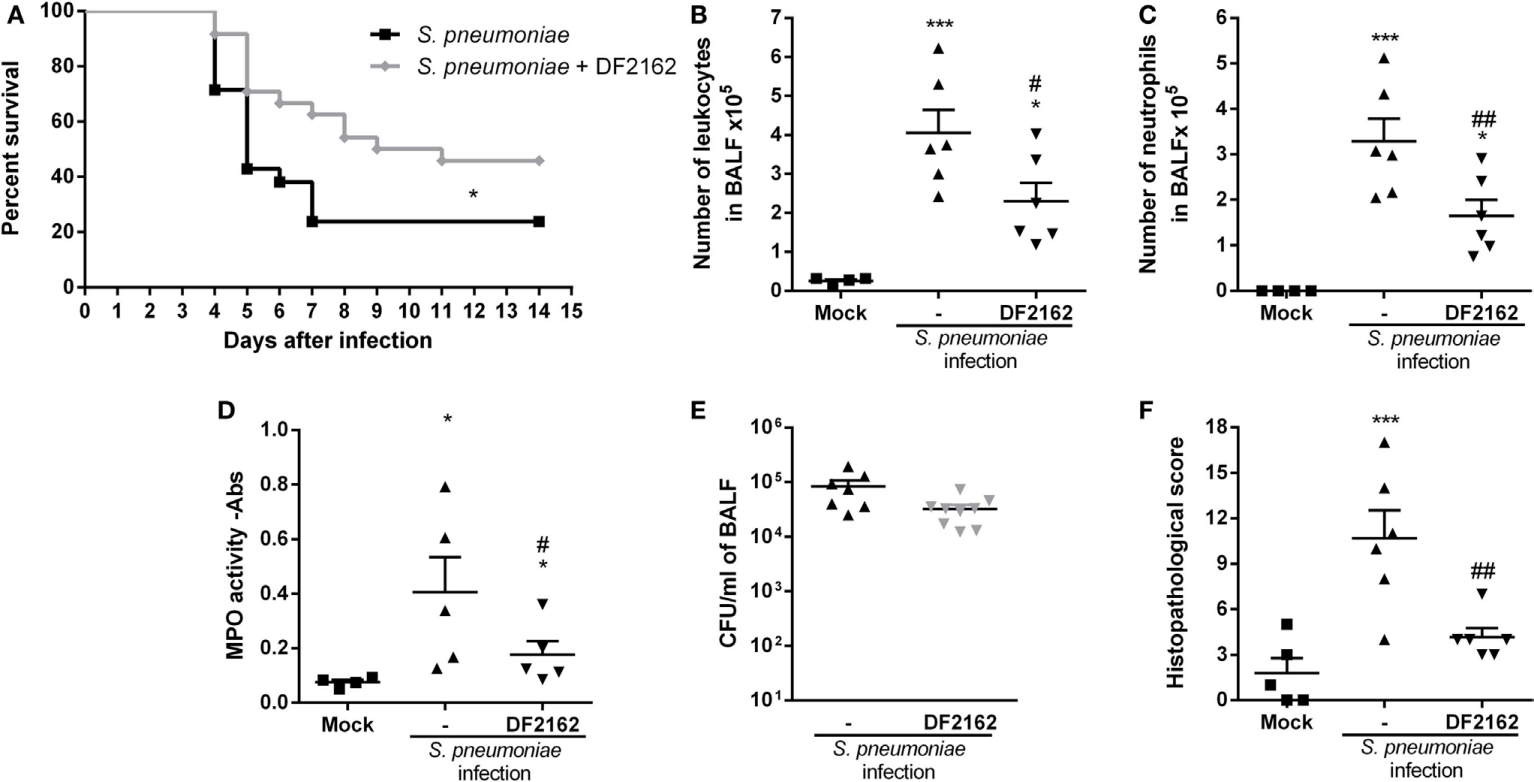
Effects of CXCR1/2 antagonism on the course of pneumococcal pneumonia in mice. Mice were infected intranasally (i.n.) with 10^4^ CFU of *Streptococcus pneumoniae* or PBS (Mock) and treated with DF2162 (10 mg/kg) twice a day during the first 2 days of infection or with the drug vehicle (carboxymethylcellulose 0.1% in PBS). For lethality, mice were accompanied daily for 10 days **(A)**. At 48 h after infection, mice were euthanized and the number of total leukocytes **(B)** and neutrophils in BALF **(C)** and myeloperoxidase assay (MPO) levels in the lungs **(D)** were accessed. Number of bacteria in BALF was also measured **(E)**. Graph **(F)** shows the overall pathological score (maximum of 18 points). Results are shown as the median **(E)** or mean ± SEM (all other graphs) of at least six mice in each group (representative of three independent experiments). *P < 0.05; ***P < 0.001, when compared with Mock group and ^#^*P* < 0.05 and ^##^*P* < 0.01 when compared to vehicle-treated group.

Due to the presence of neutrophilic inflammation in the course of bacterial lung infection with *S. pneumoniae* (13), we wondered whether treatment with the CXCR1/2 antagonist would affect pneumococcal pneumonia. Neutrophils are known to be crucial to control the replication and dissemination of bacteria but are also correlated with lung damage and death during pneumococcal pneumonia (13). Therefore, mice were treated with DF2162 from day 0—6 h after *S. pneumoniae* infection—to day 2 and lethality rates and inflammation parameters were assessed. In the context of pneumococcal infection, DF2162 treatment slightly reduced the lethality rate (Figure 4A), which seemed to be linked to decreased leukocyte, especially neutrophil, trafficking into the airways (Figures 4B,C). Importantly, despite the reduced neutrophilia in the airways and lungs (Figure 4D), short-term DF2162 treatment did not amplify the bacterial burden in the airways (Figure 4E). Furthermore, histological analysis of the lungs of infected mice showed that treatment with DF2162 reduced lung injury associated with this bacterial infection (Figure 4F; Figure S2 in Supplementary Material). Collectively, these data show that short-term DF2162 treatment reduced pulmonary inflammation caused by *S. pneumoniae* infection, an effect associated with improved survival, without affecting the ability of the host to control infection.

### CXCR1/2 Antagonism Protects from Pneumococcal Infection following IAV Infection

Secondary bacterial pneumonia, mainly caused by *S. pneumoniae*, is an important contributor for the severe prognosis of IAV-infected patients, leading to significant morbidity and mortality (19). During influenza pandemics, such as the one that occurred in 2009, a significant percentage of the fatal cases were due to secondary pneumococcal infections, despite the use of antibiotics (20–22). The exacerbated inflammatory response triggered by the secondary bacterial infection is one of the reasons for this increased mortality. Once CXCR1/2 antagonism, given before or 6 h after infection, did not decrease the ability of the murine host to deal with either influenza or streptococcal proliferation, we wondered if it would provide a therapeutic benefit to mice undergoing a secondary pneumococcal pneumonia after influenza. Therefore, we hypothesized that treatment with DF2162 during IAV infection could lead to a better outcome after a secondary pneumococcal infection.

**Figure 5:**
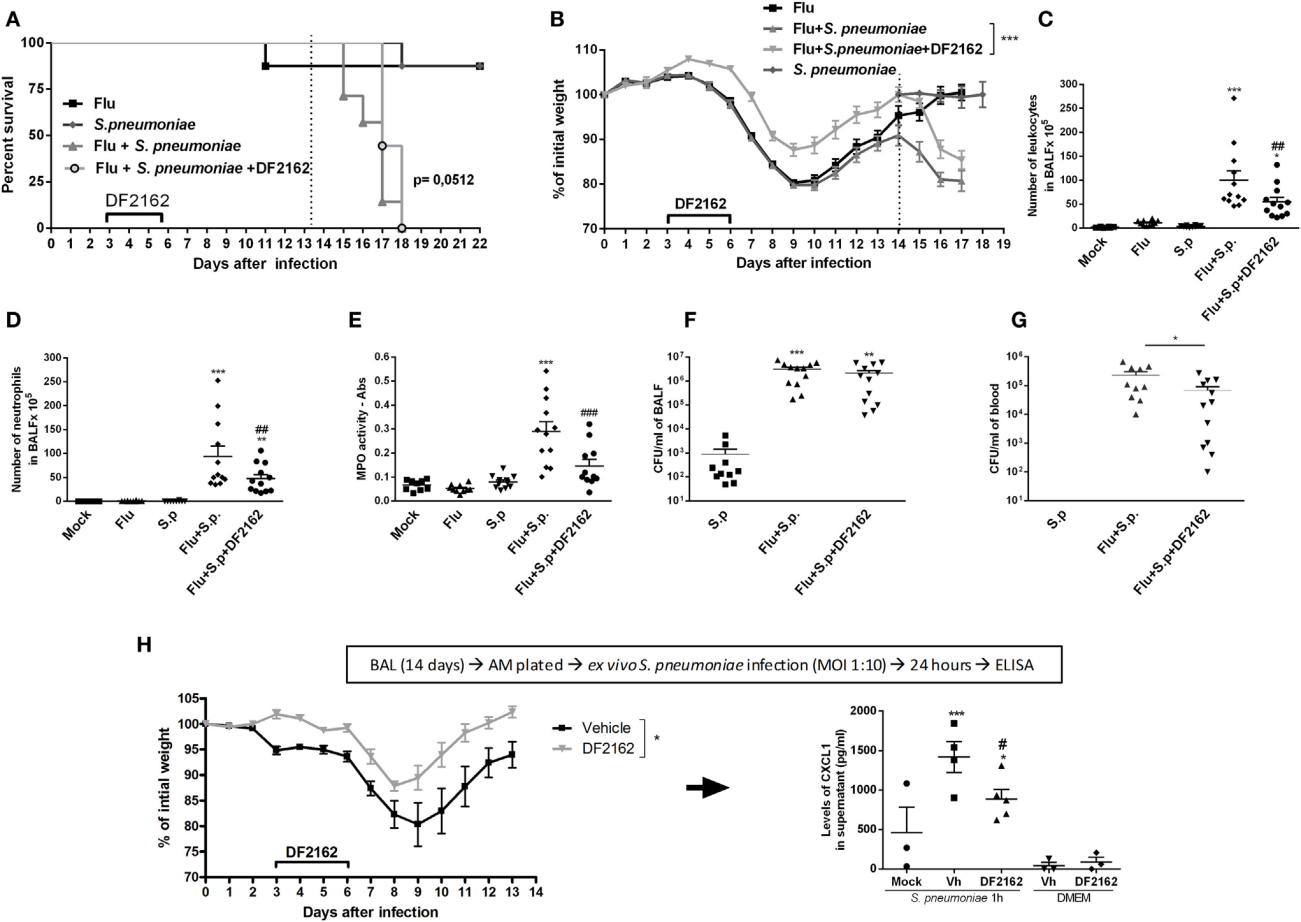
Weight loss, neutrophils recruitment, and bacteria in blood and macrophage activation of secondary infected mice are reduced after CXCR1/2 treatment. Mice were infected with influenza A virus (IAV) (500 PFU, i.n.) and after 3, 4, 5, and 6 days of infection were treated twice a day with DF2162 (10 mg/kg—oral gavage) or the vehicle of the drug. After 14 days of IAV infection, mice were secondary infected with *Streptococcus pneumoniae* (10^3^ CFU, i.n.). Single infections were also performed. Mock mice were instilled (i.n.) with PBS. The lethality [**(A)**, *n* = 7–9 mice per group, representative of three independent experiment] and weight loss [**(B)**, *n* = 20–35 mice per group, compilation of three independent experiments] were accompanied. In another experiment, mice under the same treatments and infection conditions were euthanized after 48 h after the second infection. Number of total leukocytes **(C)** and neutrophils **(D)** in the airways, neutrophils in the lungs [**(E)**— myeloperoxidase assay (MPO) assay] and bacteria in BALF **(F)** or in blood **(G)** were accessed (*n* = 4–6 mice per group, representative of two independent experiments). In another experiment, IAV-infected mice treated with vehicle or DF2162 as above were euthanized after 14 days; alveolar macrophages recovered from BALF were *ex vivo* infected with *S. pneumoniae* (MOI 1:10); after 24 h supernatant was collected for CXCL1 measurement **(H)**. The results are presented as mean ± SEM. *P < 0.05; **P < 0.01, and ***P < 0.001 when compared to Mock group; ^##^*P* < 0.01 and ^###^*P* < 0.001 when compared to Vehicle group.

To address this question, mice were infected with a lower inoculum of IAV (500 PFU) and treated with DF2162 or vehicle from days 3 to 6 after IAV infection. At day 14 after IAV infection, no virus was detected in the lungs of infected mice (data not shown). Mice were then challenged with a sublethal dose of *S. pneumoniae* (10^3^ CFU, a secondary infection). Control mice received a single infection with either IAV or *S. pneumoniae*. Both single infections resulted in mild disease with low lethality rates and small weight loss (Figures 5A,B). At day 16 after single IAV infection or at day 2 after single pneumococcal challenge (low inoculum), the number of neutrophils was similar to that found in the control uninfected group (Figures 5D,E). In addition, only a small number of bacteria could be found in the airways and no bacteria could be found in blood of mice infected with *S. pneumoniae* only (Figures 5F,G). In contrast, pneumococcal infection after IAV infection led to massive recruitment of neutrophils into the airways and lungs of mice (Figures 5C–E), overgrowth of bacteria in the airways (Figure 5F), and their dissemination to the blood (Figure 5G). This resulted in 100% lethality rates in mice subjected to the two sequential infections (Figure 5A).

In these experiments, DF2162 was administered only from days 3 to 6 after influenza infection. This is a therapeutically relevant schedule, as it is given many days after the onset of flu and before the presence of potential secondary infection. Administration of DF2162 only slightly delayed mortality after secondary infection (Figure 5A and Table 1 in Supplementary Material) and reduced the weight loss (Figure 5B). This was associated with decreased recruitment of neutrophils into the airways (Figure 5D) and lungs (Figure 5E) after the secondary infection. Once again, despite the reduction in the number of neutrophils, we observed that bacteria counts in the airways of mice were not altered (Figure 5F). Interestingly, there was reduction in number of bacteria in the blood of these mice (Figure 5G), suggesting the treatment prevented the dissemination of bacterial to blood of mice exposed to the two subsequent infections.

In a separate series of experiments, we examined whether IAV infection would enhance the ability of macrophages to secrete cytokines in response to *in vitro* infection with *S. pneumoniae*. To this end, animals were infected with IAV and treated with DF2162 or vehicle from days 3 to 6 after infection. Macrophages were then purified from the BAL of these animals at day 14 and exposed *ex vivo* to *S. pneumoniae*. As seen in Figure 5H, DF2162-treated and -infected animals had less weight loss than infected animals given vehicle. Pulmonary macrophages obtained from mice infected 14 days before with IAV clearly produced significant larger amounts of CXCL1 when exposed to *S. pneumoniae* than macrophages obtained from uninfected mice (Figure 5H). More importantly, treatment of mice from days 3 to 6 with DF2162 significantly decreased the enhanced production of CXCL1 by macrophages from mice infected 14 days before with IAV (Figure 5H).

**Figure 6:**
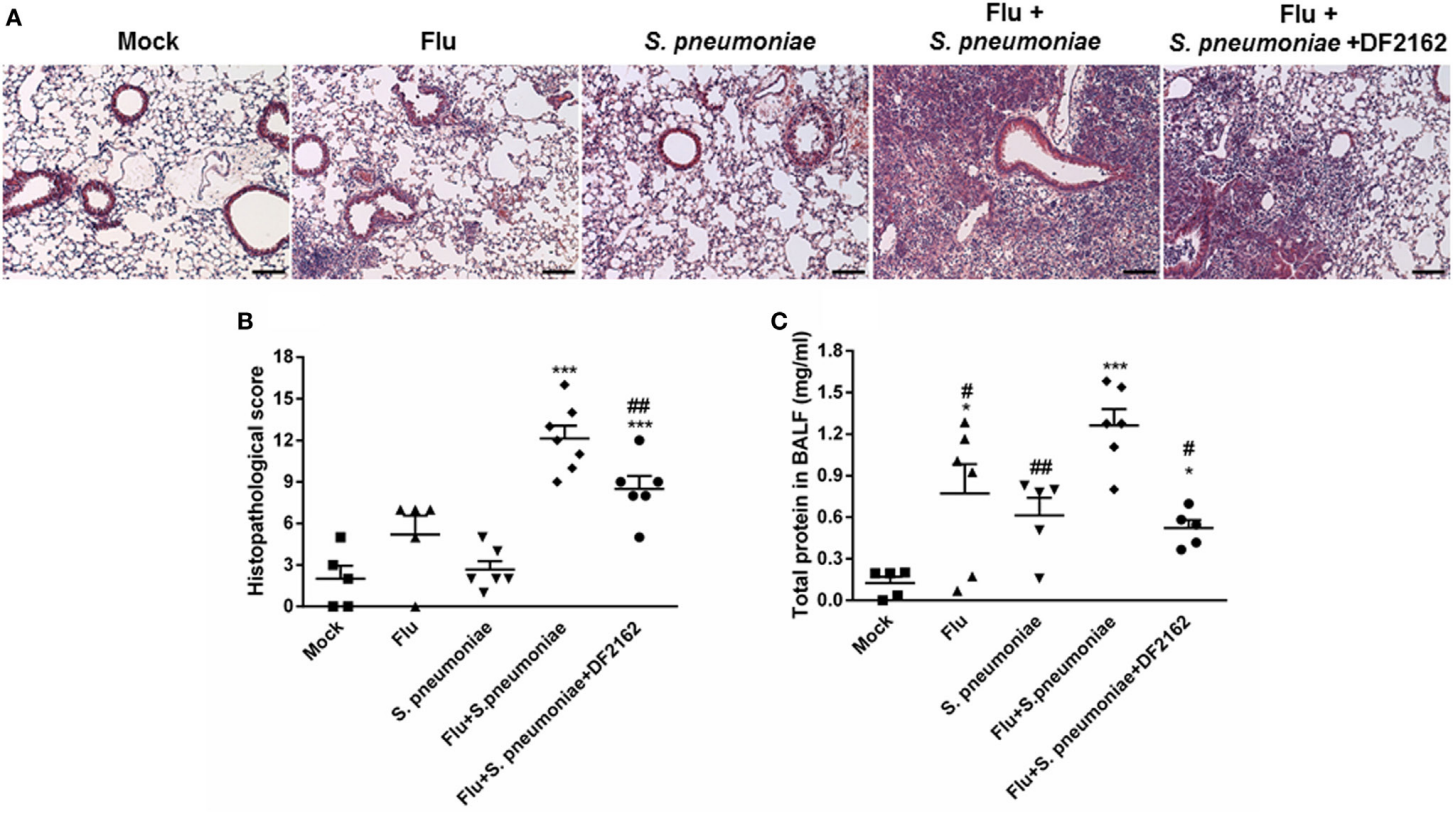
Increased lung injury due to secondary pneumococcal infection is reduced after DF2162 treatment. Mice were infected with influenza A virus (IAV) (500 PFU, i.n.) and at 3, 4, 5, and 6 days after infection were treated twice a day with DF2162 (10 mg/kg—oral gavage) or the vehicle of the drug. The animals only received the drug during the IAV infection. After 14 days of IAV infection, mice were secondary infected with *Streptococcus pneumoniae* (10^3^ CFU, i.n.). Single infections were also performed. Mock mice were instilled (i.n.) with PBS. After 48 h of secondary infection, lungs were collected, processed, and histological analysis were performed. Representative slides of Mock, single infected mice (IAV and *S. pneumoniae*), and secondary infected mice (vehicle and DF2162-treated) are shown in **(A)** (bars represent 150 µm in magnification of 100×). Graph **(B)** shows the overall score of lung injury of infected mice. Bronchoalveolar lavage fluid was used to measure the protein leakage due to infection **(C)**. Data are presented as mean ± SEM. *P < 0.05; **P < 0.01, and ***P < 0.001, when compared with Vehicle mice or indicated groups (*n* = 5–6 mice per group, representative of two independent experiments).

The decreased influx of neutrophils in mice treated with DF2162 resulted in reduction of the intense lung damage associated with secondary infection, as assessed by analysis of tissues sections of lungs of infected animals (Figures 6A,B). Single infections with sublethal inocula of IAV or *S*. *pneumonia* triggered mild airway, vascular and parenchyma inflammation, characterized by discrete leukocyte infiltrate. In contrast, infection with *S*. *pneumoniae* after IAV induced massive polymorphonuclear and mononuclear cell migration into the airways with significant loss of parenchyma architecture. The lungs of some mice presented areas of necrosis and fibrotic tissue. Treatment with DF2162 significantly decreased lung damage, as determined morphologically in tissue sections (Figure 6B). To confirm these results, levels of protein in the fluid of BAL were measured as marker of plasma leakage, and thus disruption of lung epithelial barrier or tissue injury (15). The assessment of protein leakage showed that the levels of protein in BALF were still observed in infected mice at 16 days after IAV infection. Secondary pneumococcal infection led to much higher protein leakage than single infections and, in agreement with the histological results, DF2162 treatment decreased the levels of protein in BALF (Figure 6C).

**Figure 7:**
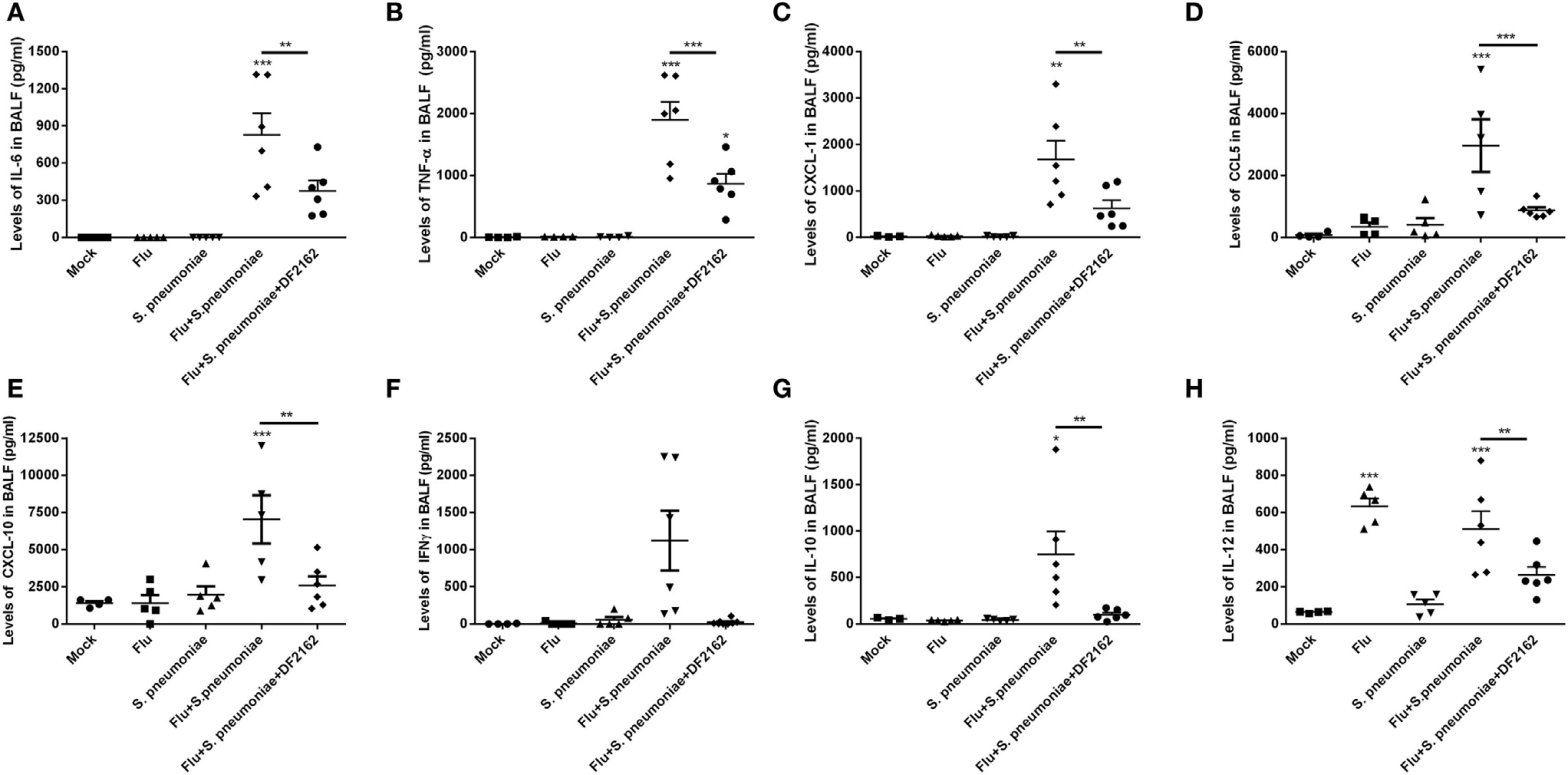
CXCR1/2 antagonism during influenza A virus (IAV) primary infection reduced the levels of cytokines during pneumococcal secondary infection. Mice were infected with IAV (500 PFU, i.n.) and at 3, 4, 5, and 6 days after infection were treated twice a day with DF2162 (10 mg/kg—oral gavage) or the vehicle of the drug. The animals only received the drug during the IAV infection. After 14 days of IAV infection, mice were secondary infected with *Streptococcus pneumoniae* (10^3^ CFU, i.n.). Single infections were also performed. Mock mice were instilled (i.n.) with PBS. After 48 h of the *S. pneumoniae* infection, mice were euthanized and the levels of IL-6 **(A)**, TNF-α **(B)**, CXCL1 **(C)**, CCL5 **(D)**, CXCL10 **(E)**, IFN-γ **(F)**, IL-10 **(G)**, and IL-12 **(H)** were measured in the BAL fluid. Data are presented as mean ± SEM. *P < 0.05; **P < 0.01, and ***P < 0.001, when compared with Vehicle mice or indicated groups (*n* = 5–6 mice per group, representative of two independent experiments).

The amelioration of tissue lesion by treatment with DF2162 was reflected in the levels of cytokines and chemokines measured in the lungs 2 days after *S. pneumoniae* infection. Indeed, as seen in Figure 7, *S. pneumoniae* infection of mice previously exposed to IAV was accompanied by massive local increase of various pro-inflammatory cytokines, including IL-6, TNF-α, CXCL-1, CCL5, CXCL-10, and IFN-γ. Levels of these cytokines were not elevated after single *S. pneumoniae* or IAV infection (Figures 7A–F). Treatment with DF2162 during the course of IAV infection greatly reduced the enhanced production of cytokines and chemokines observed 48 h after *S. pneumoniae* infection (Figures 7A–F), a result consistent with the effects of the compound on other parameters (see Figures 5 and 6). Treatment with DF2162 also prevented the increase in levels of the anti-inflammatory cytokine IL-10 (Figure 7G). Interestingly, IL-12 was the only measured cytokine that was high at day 16 after a single IAV infection (Figure 7H). *S. pneumoniae* infection alone did not result in elevation of IL-12 and *S. pneumoniae* did not affect significantly the levels of IL-12 induced by IAV (Figure 7H). In contrast, treatment with DF2162 did decrease levels of this cytokine measured after subsequent *S. pneumoniae* and IAV infections (Figure 7H).

**Figure 8:**
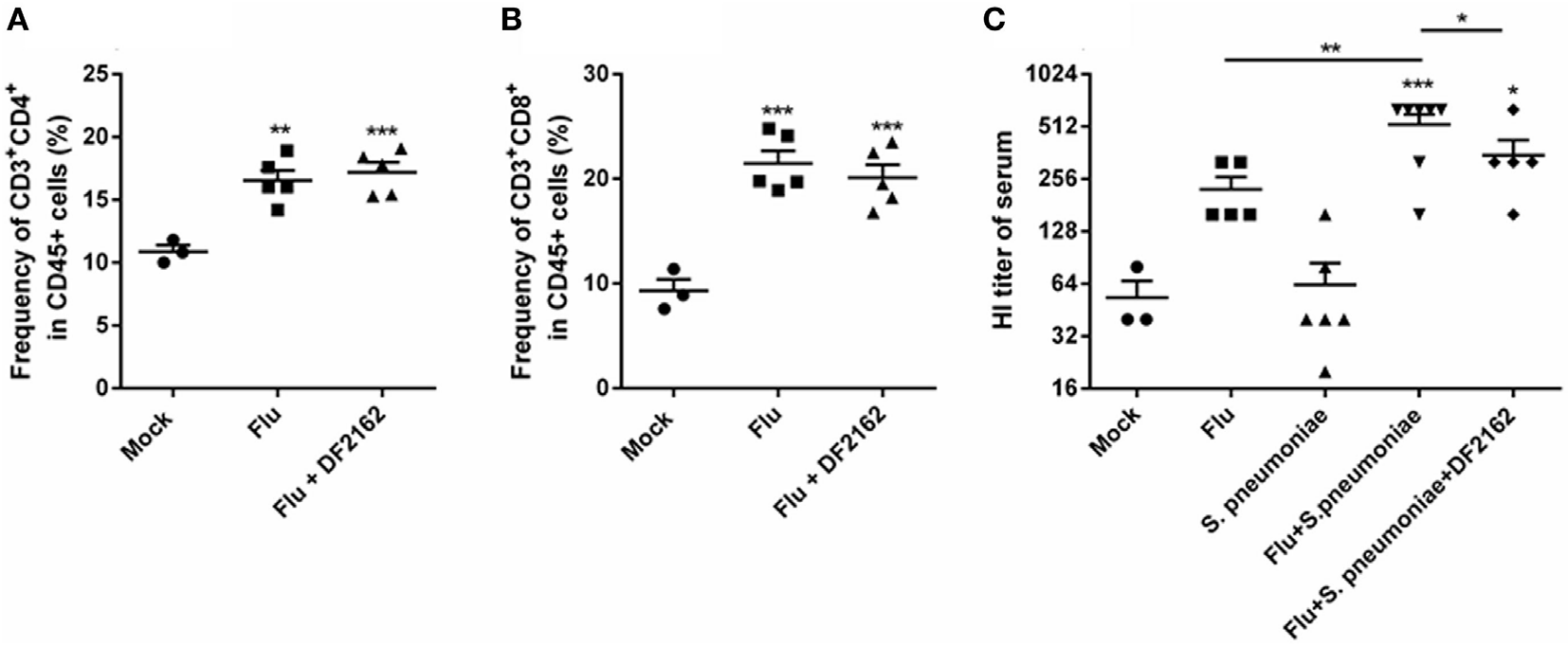
CXCR1/2 antagonism during influenza A virus (IAV) primary infection did not impair development of adaptive immune responses. Mice were infected with IAV (500 PFU, i.n.) and at 3, 4, 5, and 6 days after infection were treated twice a day with DF2162 (10 mg/kg—oral gavage) or the vehicle of the drug. The animals only received the drug during the IAV infection. After 14 days of IAV infection, mice were euthanized after 14 days of IAV infection and the lungs were collected to access the frequency of T cells—CD45^+^CD3^+^CD4^+^ **(A)** and CD45^+^CD3^+^CD8^+^ **(B)** recruited into the lungs. After 2 days of secondary pneumococcal infection at day 14 infected, mice were euthanized to assess antibodies levels **(C)** in serum. Data are presented as mean ± SEM. **P < 0.01 and ***P < 0.001, when compared with Mock mice or indicated groups and ^#^*P* < 0.05 and ^##^*P* < 0.01 when compared to Flu + *Streptococcus pneumoniae* group (*n* = 5–6 mice per group, representative of two independent experiments).

Because IL-12 is a cytokine relevant to drive Th1 responses, it was important to measure whether DF2162 would interfere with adaptive immune responses to IAV infection. To this end, animals were infected with IAV and treated with vehicle or DF2162 from days 3 to 6 after infection and samples collected on day 14. As seen Figures 8A,B, IAV infection induced a significant increase in number of CD4^+^ and CD8^+^ T cells at day 14 in the lungs of mice. Treatment with DF2162 failed to affect the accumulation of these cells. Similarly, treatment with DF2162 had no effect on systemic levels of IAV-specific antibodies (Figure 8C).

## Discussion

Acute respiratory infections (including pneumonia) are a leading cause of death in children worldwide and were responsible for approximately 15% of all deaths in 2015.^2^ Several different microorganisms are known to cause pneumonia, including fungi, bacteria, and viruses. Among the viruses, influenza virus is one of the most common causes of pneumonia. It causes seasonal epidemics leading to high number of hospitalizations every year. Novel strains of IAV may cause pandemics, with high infectivity and mortality rates. During IAV epidemics or pandemics, there is a high correlation between increased mortality and secondary bacterial infections (23, 24). *S. pneumoniae* is the leading cause of community-acquired pneumonia and the most common pathogen secondary to a previous IAV infection (19). Secondary pneumococcal infections are characterized by exacerbated inflammatory responses with massive number of neutrophils recruited into the lungs. Despite the increased number of immune cells in the lungs, during pneumococcal pneumonia following flu, the number of bacteria in lungs and in blood is high and intense lung injury is observed. Therefore, the uncontrolled inflammation appears to be harmful rather than protective. Indeed, the lack of negative regulators of inflammation, such as ST2, has been shown to aggravate secondary pneumococcal infection leading to higher mortality and number of bacteria in the lungs of infected mice (25). Antibiotic treatment, mainly β-lactams, is recommended during secondary pneumococcal pneumonia (26) but can also aggravate the inflammatory response leading to worse disease outcome. Bacteria lysis, as a consequence of antibiotic treatment, results in overactivation of the immune system by the release of pathogen-associated molecular patterns and therefore can exacerbate the inflammatory response (27). Indeed, treatment with antibiotics did not improve patient survival in severe cases of secondary pneumonia following flu (20, 28). In this context, adjunctive treatment with corticosteroids has been suggested as a strategy to control the overwhelming inflammatory response and decrease the associated immunopathology (29).

This study investigated the role of CXCR1/2 during influenza and pneumococcal infections. The major findings of this study can be summarized as follows: CXCR1/2 antagonism during IAV infection (i) improved morbidity, decreased neutrophilic inflammation, decreased the viral titer in the lungs, and reduced the immunopathology associated with IAV infection of mice; in addition, it could also (ii) slightly decrease lethality during primary pneumococcal infection, which was associated with less inflammation and lung injury. We also showed that (iii) IAV infection increased susceptibility of mice to a secondary pneumococcal infection and this was associated with a massive inflammatory response, overgrowth and dissemination of bacteria to bloodstream, and intense lung damage. In this scenario, treatment with DF2162 during IAV infection (iv) reduced the intensity of the neutrophilic inflammation and the levels of pro-inflammatory cytokines, decreased the number of bacteria in the blood of infected mice, and partially prevented the lung damage associated with the secondary pneumococcal pneumonia. Importantly, DF2162 treatment (v) did not impair the development of the adaptive immune responses after IAV infection. Therefore, these data show the important role of the exacerbated inflammatory responses in mediating morbidity during respiratory infections by IAV and *S. pneumoniae*. Moreover, the data show that CXCR1/2 antagonism clearly decreased inflammation association with the infection and resulted in a more favorable outcome.

Neutrophils are rapidly recruited into the lungs in response to either IAV or *S*. *pneumoniae* infection. Neutrophil recruitment is coordinated by the production of chemokines that bind to receptors on the surface of leukocytes. CXCR1/2 is known to be a major receptor for the recruitment of neutrophils in various models of neutrophilic inflammation, including in the lung (30, 31). CXCR1/2 binds to and signals in response to CXCL8 and other ELR + CXC chemokines (11). During IAV infection, neutrophilic inflammation precedes the mortality of mice (15) and the intensity of the neutrophil influx is correlated with the higher lethality, as observed in mice infected with a more severe inoculum of virus (10^6^ PFU—100% of mortality after 9 days) (15). Indeed, we and others have shown that the inflammatory responses following IAV infection are associated with increased lung damage that resulted in increased morbidity and mortality (15, 32).

Because CXCR1/2 drives neutrophil influx in experimental models and neutrophils may contribute to the pathogenesis of certain inflammatory diseases, several CXCR1/2 antagonists, including DF2162, have been developed (33). These antagonists are effective at blocking chemokine-driven neutrophil influx in various models of inflammation and some are undergoing clinical evaluation in humans (34, 35). Here, we have used the CXCR1/2 antagonist DF2162 because of its suitable pharmacokinetic profile and effectiveness in murine models of neutrophilic inflammation (14, 16).

The effects of CXCR1/2 antagonism in the context of IAV or *S. pneumoniae* infections is controversial. It has been shown that during IAV infection, CXCR2 is very important for the recruitment of neutrophils into the lungs but is not necessary for viral clearance (36). A different study showed that CXCL2 production by alveolar epithelial cells in transgenic mice encoding IAV hemagglutinin in epithelial cells was associated with lung injury and the lethality of mice (37). CXCR2-deficient mice had less lung injury and lethality after a challenge with influenza-specific CD8^+^ T cells (37). In addition, neutralization of CXCR2 with antibodies was shown to reduce inflammation and associated lung injury and improved survival in mice deficient in the regulatory molecule IRAK-M (38). In agreement with these studies, our work shows that CXCL1 and CXCL2 are produced in high amounts in the early stages of infection and this was correlated with the increased neutrophil recruitment into the airways and lungs of infected mice. The intensity of production of these chemokines and the recruitment of neutrophils was proportional to the morbidity and mortality of mice (comparing 10^4^ and 10^6^ PFU inocula). In our experiments, blockade of CXCR1/2 decreased inflammation and morbidity during IAV infection but had a small effect on viral clearance. In contrast to the latter and our findings, another study showed that the complete absence of CXCR2 did not result in improvement of lethality or morbidity after IAV infection (36). This could be partially explained by the different strategies used—CXCR2 knockout strategy and the pharmacological inhibition of this receptor. In the gene-deficient animal, there is total absence of the receptor and this may lead to a more intense phenotype. Pharmacological treatments usually do not block receptor occupancy by 100% but there is a risk of off-target effects (low specificity). DF2162 is a very selective allosteric compound that does not displace the ligand (CLXCL1 or 2, in mice) but appears to act *via* binding to an allosteric site at the chemokine receptor (14). Thus, whereas an off-target effect appears less likely, DF2162 may not prevent completely signaling by triggering CXCR1 or CXCR2. Indeed, DF2162 blocks CXCR1 in addition to CXCR2, and not only CXCR2 as in CXCR2-deficient mice, but it is unlikely that double inhibition would account for the greater phenotype of CXCR2-deficient mice. There have been very few studies with mice that lack the CXCR1 receptor, so that the real role of this receptor in this species is not known. Regardless of the reason explaining the difference of response between our study (using drugs) and another (using gene-deficient mice) (36), it is clear that treatment of patients is based on pharmacological agents with their limitations and potential advantages, as suggested here.

Interestingly, the antagonism of CXCR1/2 did not inhibit the accumulation of neutrophils into pulmonary parenchyma. Therefore, the decreased number of neutrophils in the airways of infected animals treated with DF2162 was sufficient to decrease lung damage due to excessive neutrophil recruitment/activation. Indeed, it has already been shown that reduction in the number of neutrophils in the airways, but not in the lung parenchyma, could reduce morbidity and mortality of mice after IAV infection (14). Therefore, although neutrophils are crucial for the ability of the murine host to cope with IAV infection, the partial blockade attained with CXCR1/2 antagonists preserves the host immune response to infection but decreases the exacerbated pulmonary inflammation that causes morbidity.

A similar phenotype was observed in a model of *S. pneumoniae* infection. Despite the reduction in the number of neutrophils in the lungs of pneumococcus-infected mice, there was no impairment in the clearance of bacteria, as seen by the same number of CFU in the airways of both vehicle-treated and DF2162-treated mice. This is consistent with other studies that showed that reduction in neutrophil recruitment led to better outcome from pneumococcal pneumonia in mice (13, 39). Treatment with a CXCR2 antagonist could also prevent mice mortality in a murine model of pneumococcal meningitis (40). On the other hand, another study showed that pretreatment with SB-225002, a CXCR2 antagonist, increased bacteria burden and showed that CXCR2 was essential to protect mice from pneumococcal pneumonia (41). In addition, CXCR2 KO mice were shown to be more susceptible to pneumococcal lung infection. In our experiments, DF2162 treatment was not given prophylactic as in the former studies (42), but it was started after infection. It has been suggested that neutrophils are more important in the first hours of pneumococcus infection, but then they may become detrimental to the host later (39). Therefore, it is possible that our treatment schedule allowed these cells to act against the pathogen, without harming the host. In addition, in the context of experiments in which *S. pneumoniae* infection followed IAV infection, the treatment with DF2162 was given during the period of viral infection, hence many days before the bacterial infection.

Influenza A virus infection is a known cause of the increase in susceptibility to secondary bacterial infections. Several mechanisms have been described to play a role in this process, including direct lung damage caused by the virus that would lead to decreased beating of ciliary tracheal epithelial cells (42), increased neuraminidase activity (43), and an unregulated immune response (44). During secondary pneumococcal infection, there is a massive influx of neutrophils and production of cytokines in the airways and lungs of infected mice. Despite the great increase in number of cells and pro-inflammatory molecules, overgrowth and dissemination of bacteria are observed during secondary infection. This has led to the hypothesis that there is impairment on function of neutrophils during secondary infection (45). It is suggested that the exacerbated number of neutrophils cannot control bacteria proliferation while contributing to the increased lung injury and loss of lung function during secondary pneumococcal infection. Indeed, we observed that 14 days after IAV infection, the secondary pneumococcal infection led to substantial influx of neutrophils into the lungs and greater proliferation of bacteria and further dissemination to the blood. Consequently, severe lung damage and increased mortality was observed. Thus, it is possible that the reduction of the number of neutrophils after DF2162 treatment could account for the decreased lung injury associated with the secondary infection. The decreased recruitment of neutrophils could implicate in less production of oxygen reactive species, proteases, and neutrophil extracellular traps (NETs) in the lungs. Overproduction of reactive oxygen species following flu has been shown to be an important contributor for the worse outcome from disease once it increases the apoptosis of pulmonary epithelial cells and edema, contributing to the intense lung injury following IAV infection (46). In addition, the higher activation of neutrophils during IAV infection can lead to increased production of NETs, which are also dependent on redox enzyme activities. These have been shown to be associated not only with lung injury during IAV single infection but have also been implicated in mediating pulmonary dysfunction in the context of secondary bacterial infection (47). Therefore, the control of neutrophil recruitment and activation by DF2162 could lead to decrease in production of several molecules and processes that eventually may result in lung injury.

It has been shown that during severe respiratory infections, the production of pro-inflammatory cytokines is proportionally associated with the severity of disease (48, 49). The massive production of cytokines such as TNF-α, IL-6, IL-12, CXCL1, and CXCL2 was a hallmark of the secondary pneumococcal infection after the flu. The increased production of these molecules may lead not only to excessive recruitment of neutrophils but also to overactivation of these cells, hence inducing intense production of proteases, reactive oxygen species, and NETs that could directly cause pulmonary damage. Moreover, these cytokines may cause overactivation and eventual apoptosis of other pulmonary cell types including epithelial and endothelial cells. A consequence of activation and apoptosis of epithelial cells would be loss of barrier function. CXCR2 is also expressed by endothelial cells and is known to play a role in vascular permeability and increased edema formation (50–52). Activation of neutrophils in the lung may also contribute to edema formation. In animals treated with DF2162, there was a decrease in production of pro-inflammatory mediators and protein leakage as compared to mice that were infected with pneumococcus after IAV infection.

In terms of anti-inflammatory cytokines, we and others have shown that levels of IL-10 were significantly increased after secondary pneumococcal infection (53). This was suggested to be associated with the inability of the cells to deal with infection, despite the exacerbation of inflammation and massive recruitment of neutrophils (54, 55). Therefore, reduction of IL-10 during a secondary pneumococcal infection may be beneficial for the host (55). In agreement with these data, DF2162 decreased the levels of IL-10 in the airways of infected mice what could contribute to the better outcome from infection, and no impairment in bacteria control.

Inflammation is known to play a dual role during infection—it is extremely important to control pathogen dissemination but it may have collateral damage and lead to intense tissue and worse prognosis during infection. It has been suggested that taming aspects of inflammation that contribute to tissue damage may be beneficial to the host (15). On the other hand, inflammation itself is important for the orchestration of adaptive immune responses. In this regard, one of the concerns of using DF2162 as a strategy to control inflammation was that it could impair features of the adaptive response against IAV. However, we showed that DF2162-treated mice had similar and robust response in terms of lymphocyte recruitment and antibody production when compared with IAV-infected untreated animals. Altogether, the current results show that the chemokine receptors CXCR1/2 have a major role in driving pulmonary inflammation and damage in the context of severe IAV and pneumococcal infections. Inhibition of CXCR1/2 prevented this exacerbated response, even after sequential IAV and pneumococcal infection without compromising adaptive responses against the virus. Therefore, inhibition of CXCR1/2 should be considered further as an adjunct therapy for the treatment of severe pulmonary infections caused by IAV and pneumococcus.

## Ethics Statement

All procedures described in the study had prior approval of the local animal ethics committee—Comitê de Ética em Experimentação Animal da Universidade Federal de Minas Gerais—CETEA/UFMG 13/2010 and 381/2015.

## Author Statement

We present data on the use of DF2162, a CXCR1/2 antagonist in murine models of influenza A, *Streptococcus pneumoniae*, and post-influenza pneumococcal infection. DF2162 reduces inflammation, morbidity, or mortality but does not reduce the ability of mice to control the pathogens. More importantly, the therapeutic use of DF2162 after influenza controls secondary *Streptococcus pneumoniae* infection and inflammation and may represent a promising therapeutic strategy for treating lung infections caused by these pathogens.

## Author Contributions

LT, CG, FT, and MT designed research, analyzed data, and wrote the paper. LT, CG, MM, CQ-J, and AB performed experiments and analyzed data. AM provided influenza A virus strain. AM, LS, LB, MA, and FT contributed to manuscript revision.

## Conflict of Interest Statement

The authors declare no conflict of interest. Dompé farmaceutici spa contributed to the research by providing DF2162 compound. The company has interests in the development of CXCR1/2 inhibitors for the treatment of IL-8-associated pathologies.

## Footnotes

^1^http://www.who.int/mediacentre/factsheets/fs211/en/.

^2^http://www.who.int/mediacentre/factsheets/fs331/en/.

## Supplementary Material

Figure S1 Histopathological changes in the lungs of IAV-infected mice are reduced after DF2162 treatment. Mice were infected with 10^4^ PFU of IAV and treated with DF2162 (10 mg/kg) twice a day during the first 5 days of infection or with the drug vehicle (carboxymethylcellulose 0.1% in PBS). Control animals were instilled intranasally with PBS (Mock). Representative H&E stained slides of lungs of Mock and IAV-infected animals (vehicle and DF-treated) are shown—100× magnification.

Figure S2 Treatment with CXCR1/2 antagonist prevents histopathological changes in the lungs of *Streptococcus pneumoniae*-infected mice. Mice were infected intranasally with 10^4^ CFU of *S. pneumoniae* or PBS (Mock) and treated with DF2162 (10 mg/kg) twice a day during the first 2 days of infection or with the drug vehicle (carboxymethylcellulose 0.1% in PBS). Representative H&E stained slides of lungs of Mock and *S. pneumoniae*-infected animals (vehicle and DF-treated) are shown—100× magnification (*n* = 5–6 mice per group, representative of two independent experiments).

Table S1 Mice were infected with IAV (500 PFU, i.n.) and after 3, 4, 5, and 6 days of infection were treated twice a day with DF2162 (10 mg/kg – oral gavage) or the vehicle of the drug. After 14 days of IAV infection, mice were secondary infected with *S. pneumoniae* (10^3^ CFU, i.n.). Single infections were also performed. Mock mice were instilled (i.n.) with PBS. The lethality was accompanied. Table 1 shows the raw data regarding the lethality study performed.
