# Supplementary figures and images for: CXCR1/2 Antagonism Is Protective during Influenza and Post-Influenza Pneumococcal Infection
Source: Front Immunol. 2017 Dec 13;8:1799. doi: 10.3389/fimmu.2017.01799 (PMC5733534; doi:10.3389/fimmu.2017.01799)

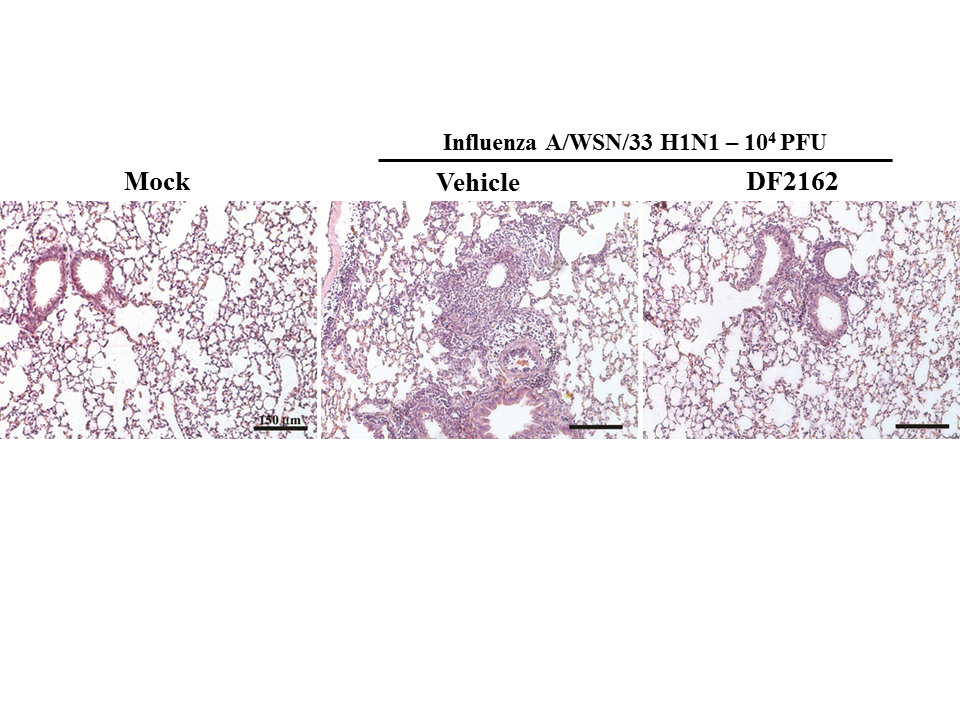

Supplement: Figure S1 — Histopathological changes in the lungs of IAV-infected mice are reduced after DF2162 treatment. Mice were infected with 104 PFU of IAV and treated with DF2162 (10 mg/kg) twice a day during the first 5 days of infection or with the drug vehicle (carboxymethylcellulose 0.1% in PBS). Control animals were instilled intranasally with PBS (Mock). Representative H&E stained slides of lungs of Mock and IAV-infected animals (vehicle and DF-treated) are shown—100× magnification. [file Image_1.TIF]

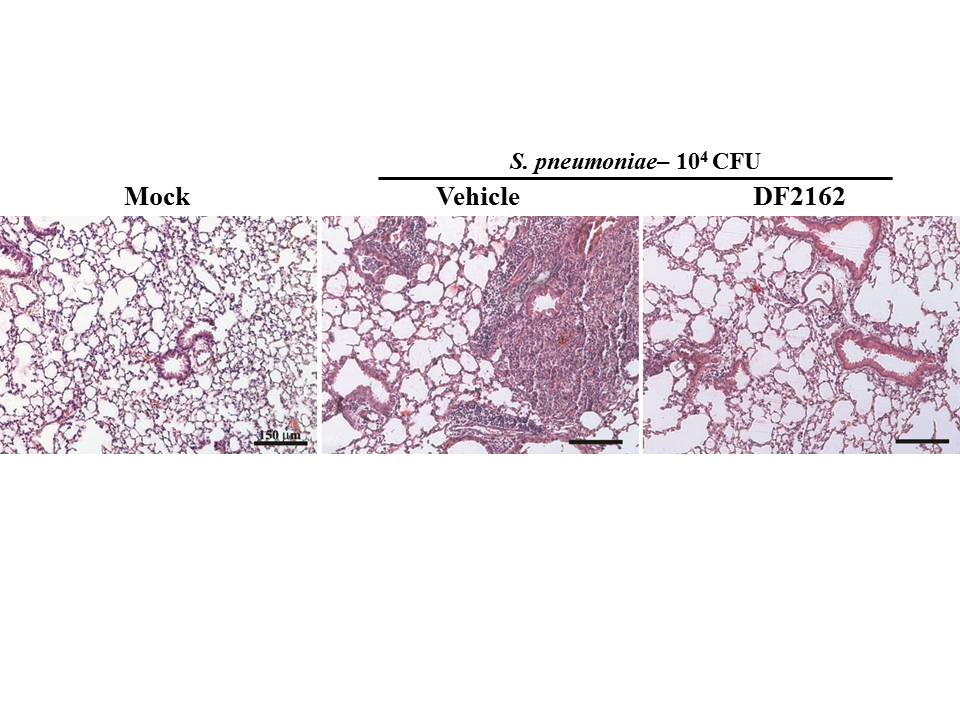

Supplement: Figure S2 — Treatment with CXCR1/2 antagonist prevents histopathological changes in the lungs of Streptococcus pneumoniae-infected mice. Mice were infected intranasally with 104 CFU of S. pneumoniae or PBS (Mock) and treated with DF2162 (10 mg/kg) twice a day during the first 2 days of infection or with the drug vehicle (carboxymethylcellulose 0.1% in PBS). Representative H&E stained slides of lungs of Mock and S. pneumoniae-infected animals (vehicle and DF-treated) are shown—100× magnification (n = 5–6 mice per group, representative of two independent experiments). [file Image_2.TIF]
